# Long-term outcome of smear-positive tuberculosis patients after initiation and completion of treatment: A ten-year retrospective cohort study

**DOI:** 10.1371/journal.pone.0193396

**Published:** 2018-03-12

**Authors:** Mesay Hailu Dangisso, Endrias Markos Woldesemayat, Daniel Gemechu Datiko, Bernt Lindtjørn

**Affiliations:** 1 Hawassa University, College of Medicine and Health Sciences, School of Public Health, Hawassa, Ethiopia; 2 University of Bergen, Faculty of Medicine, Centre for International Health, Bergen, Norway; 3 HHA - REACH ETHIOPIA Project, Hawassa, Ethiopia; 4 Liverpool School of Tropical Medicine, Liverpool, United Kingdom; Centro de Investigação em Saúde de Manhiça, MOZAMBIQUE

## Abstract

**Background:**

The status of tuberculosis (TB) patients since initiation of treatment is unknown in South Ethiopia. The objective of this study was to assess the long-term outcomes of smear-positive TB patients since initiation and completion of treatment, which includes TB recurrence and mortality of TB patients.

**Methods:**

We did a retrospective cohort study on 2,272 smear-positive TB patients who initiated treatment for TB from September 1, 2002—October 10, 2012 in health facilities in Dale district and Yirgalem town administration. We followed them from the date of start of treatment to either the date of interview or date of death.

**Results:**

Recurrence rate of TB was 15.2 per 1000 person-years. Recurrence was higher for re-treatment cases (adjusted hazard ratio (aHR), 2.7; 95% CI, 1.4–5.3). Mortality rate of TB patients was 27.1 per 1,000 person-years. The risk was high for patients above 34 years of age (aHR, 2.1; 95% CI, 1.2–3.9), poor patients (aHR, 1.3; 95% CI, 1.0–1.8), patients with poor treatment outcomes (aHR, 6.7; 95% CI, 5.1–8.9) and for patients treated at least 3 times (aHR 4.8; 95% CI, 2.1–11.1). The excess mortality occurred among patients aged above 34 years was high (41.2/1000 person years).

**Conclusion:**

High TB recurrence and death of TB patients was observed among our study participants. Follow-up of TB patients with the risk factors and managing them could reduce the TB burden.

## Introduction

Tuberculosis (TB) is a disease of public health concern causing considerable mortality, particularly in high TB burden areas, while most deaths are preventable [1]. In 2016, incidence rate of TB in the Africa region was 254/10^5^ and about 417 thousands people died due to TB [1]. Ethiopia is one the countries with highest TB burden, with approximately 30,000 people who died of TB and an incidence of 177/10^5^ in 2016 [1]. In a ten-year retrospective trend analysis, 3% of the TB cases died during treatment in southern Ethiopia [2]. Similar measure was 3.7% in Addis Ababa and it was 7.4% in Dangila district in Northwest Ethiopia [3, 4]. Most deaths (56.7%) of TB patients occur during the first two months of treatment [4].

Pulmonary TB causes mild to severe lung impairment, which increases with recurrences [5, 6] and this may results in death of the patient. Mortality of TB cases during treatment could be underestimated because of limited information on persons lost to follow-up during TB treatment, and non-evaluated cases [7]. Some studies reported high mortality of TB patients and high recurrence of TB among patients who successfully completed treatment [8, 9]. In southern Ethiopia, about 4% of smear-positive patients had recurrence of TB and about 9% of successfully treated TB patients died [10, 11]. Recent studies reported that death rates of TB patients during or after completing treatment ranged from 2–23% [3, 7, 12–17]. The post-treatment mortality of TB patients was 4–6 times higher than the death rate in the general population [7, 11, 17].

TB recurrence is more common in HIV patients [18, 19], patients living in neighborhoods with high TB incidence [19] and in patients who did not comply with self-administered treatment [18]. High mortality of TB cases was related to male sex, the elderly and non-farmers [11]. Another study also reported that older age contributed to an increased risk of TB death [20]. In contrary to this, younger patients had higher mortality in Chenai, India [17]. Clinical factors like smear-positivity, poor treatment outcome, multidrug-resistant TB (MDR-TB), late treatment start, history of previous treatment and comorbidity increased the risk of TB death [15, 17, 21–23]. Other studies also reported that poor nutrition and behavioral factors like smoking, alcoholism and drug abuse increased the risk of death due to TB [17, 21, 23].

High smear-positive TB recurrence and mortality of TB patients after successful treatment were reported in the southern Ethiopia region [10, 11]. However, the studies did not report the status of TB patients since the initiation of TB treatment. Generating information on the health status of TB cases at different times after diagnosis could help us to know the status of persons lost to follow-up TB treatment and TB cases whose treatment outcome was unknown. Therefore, in this study, we estimated the recurrence of TB, mortality rate and excess general mortality of smear-positive TB patients. The objective of the study was to assess the long-term outcomes of smear-positive TB patients after initiation of treatment, which includes death of TB patients and recurrent TB.

## Methods

### Study setting

The study was carried out in Dale district and Yirgalem town administration of Sidama Zone in southern Ethiopia. The Sidama Zone is one of the densely populated Zones in the region with a population of over 3.8 million. The Zone is divided in to 19 districts and four town administrations. Dale district consists of 36 rural kebeles while Yirgalem town administration has 7 urban kebeles. Kebele is the smallest administrative unit in Ethiopia with an average population of 5,000 people. We included all smear-positive TB patients in the 43 kebeles in the study.

In 2003, Ethiopia launched health extension program (HEP), a community-based initiative to improve access to primary healthcare [24]. High school completed women selected from each kebele, receive one-year training and they are deployed as health extension workers (HEWs) in their respective kebeles [25]. The HEWs provide basic primary healthcare including TB prevention and care such as delivering health education, identifying and referring presumptive TB cases, tracing persons lost to follow-up TB treatment and ensuring treatment adherence. Directly observed treatment short-course (DOTS) was primarily delivered in hospitals and health centers in the study area. Since October 2011, the Zonal health department started a community-based TB case finding and treatment to improve TB care in the Zone. The HEWs were involved in identifying patients with symptoms suggestive of TB, preparing smears and providing treatment at community level [26].

### Study design and population

We did a retrospective cohort study which consists of record review and community based survey of patients treated for TB. The outcome variables were recurrence of acid fast bacilli (AFB) positive pulmonary TB and death in previously treated smear-positive TB patients. Recurrent TB cases were those with the history of successful treatment for TB and developed again an active TB.

**Fig 1:**
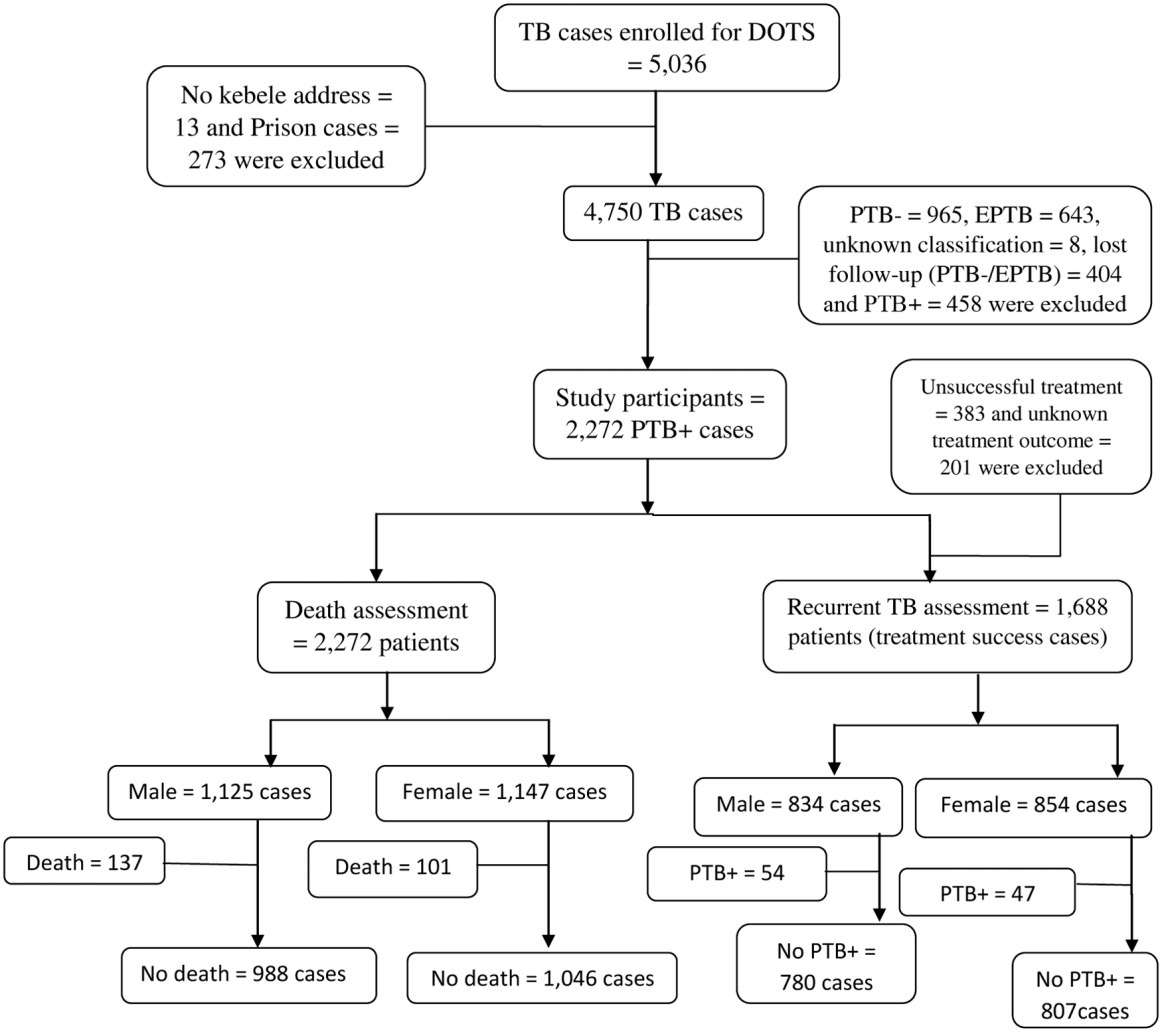
Cohort flow chart of TB patients participated in the study, Dale, South Ethiopia. DOTS: Directly Observed Treatment Short course; PTB+: Smear-positive TB; PTB-: Smear-negative TB; EPTB: Extra pulmonary TB.

The study population included all smear-positive TB cases initiated and completed treatment between September 1, 2002 and October 10, 2012 in all health facilities providing DOTS in the study area. During the study period 5,036 persons with all forms of TB were registered for treatment. Prison TB cases, patients with unknown kebele address and TB patients whose location was unknown due to migration to other areas were not interviewed. We also excluded smear-negative TB cases, extra pulmonary TB (EPTB) cases and TB cases with unknown TB classification from this report. Thus, our study is based on 2,272 smear-positive TB cases. Detail of the study profile is described in Fig 1.

### Definitions

The term TB recurrence was used to describe a recorded (on TB registry) re-diagnosis of TB after successful completion of DOTS. We confirmed the re-diagnosis through interview. Death was defined as smear-positive TB patient who died for any reason during the course of treatment or after completing TB treatment. Death after completing treatment was ascertained by interviewing any adult member in the family of the deceased person. TB cases and the DOTS treatment outcome were defined according to the national guideline [27].

### TB diagnosis and treatment

TB patients were diagnosed and treated based on the recommendations of National TB control guideline of Ethiopia [27]. Smear-positive TB cases were diagnosed by sputum microscopy which was done in health centers or in hospitals [24, 27]. The health centers perform sputum microscopy, treatment and referral of smear-negative and EPTB cases to hospitals, while hospitals diagnose and treat all forms of TB cases and they also provide inpatient services [24, 27]. The health posts support TB prevention and care through the HEWs.

Smear-positive TB cases are patients with at least two initial sputum examinations positive for AFB, or with one initial smear-positive for AFB and culture positive or with one initial smear-positive for AFB and radiographic evidence suggestive of TB [27].

### Data collection

Using unit TB registries, we registered TB cases treated during the study period, in all health facilities providing DOTS. TB cases from other districts enrolled for treatment in Dale district and in Yirgalem town administration were excluded from the study. We prepared a list of TB cases registered for treatment for each kebele. Then, enumerators went to the kebeles to identify the TB cases and interviewed them. Both data (record review and interview) were collected from September 2012 to March 2013.

Data concerning age, sex, address, treatment category, treatment times, date of treatment started, date of treatment completed and treatment outcome were collected from the TB registry. Data on education, family size and household wealth related variables were obtained through interviews. Recurrence of TB and death during TB treatment or after completion of treatment were identified both from the registry and by the interview.

Enumerators were university graduates and we recruited guiders from the local community who knew houses of the patients. Guiders were used to locate the address of TB cases. The data collectors interviewed the TB cases at their residences if they were alive. Any family members of the cases were interviewed if the TB cases had died.

Pretested and structured questionnaire was used for data collection. We trained the data collectors and supervisors on the data collection formats. Data collection process was supervised on a daily basis. We checked the consistency of collected data with the information in the TB registries. To ensure the completeness and accuracy of the data, the number of cases and patient information entered in each year and health facility were checked page by page and by the year of treatment with the information in the TB registry. Data were double entered in to Microsoft access and then cross-checked for consistency of the two versions. During the preliminary analysis we looked for errors and corrected them by re-checking the TB registries.

### Statistical analysis

Data were analyzed using SPSS 20 (SPSS, Inc., Chicago, IL) statistical software. We used descriptive statistics to summarize socio-demographic characteristics and clinical information of the study participants. TB recurrence rate was calculated as the number of recurrent TB cases per 1,000 person-years. Mortality rate was calculated as the number of deaths per 1,000 person-years. Case fatality rate (CFR) was measured by dividing the number of TB cases died during the follow-up period per 100-population at risk in the beginning of the study. For death rate, person-year of observation was calculated from the date of starting TB treatment to the date of death if the patient died or to the date of interview (last date of observation). Last date of observation considered in this study was October 10, 2012. For TB recurrence rate estimation, person-year of observation was calculated from the date of completing treatment to the date of interview (last date of observation). The study outcomes were censored if the patients were reported to be dead or had recurrent TB at any time during the study period.

We calculated frequencies. A Kaplan-Meier plot was used to estimate survival probability by age, treatment outcome, treatment times and wealth index. Cox’s proportional hazards model was used to determine risk factors of TB recurrence and risk factors of death. Variables with P value of less than 0.2 in the bivariate analyses were included in the multivariate cox’s proportional hazards model. Adjusted hazard ratio (aHR) and the corresponding 95% confidence interval were determined.

To compute the standardized mortality ratio (SMR), we compared the mortality rate among our study participants to the mortality rate in the standard population. The standard mortality rate used for this study was mortality rate of the general population in the Sidama Zone, reported by the Central Statistics Agency (CSA) of Ethiopia for 2007 [28]. We measured excess mortality by subtracting age, sex and address specific mortality in the reference population from mortality among smear-positive TB patients initiated DOTS.

We did a principal component analysis to construct a household wealth index. Nine household wealth related variables were included in the analysis as recommended by Vias S et al and Howe LD [29, 30]. These were type of housing, type of floor, availability of window for the house, having radio, presence of television, having motor cycle, availability of animal drawn cart, having any domestic animals and availability of cash crop in the household. We used similar analysis in our previous work [31]. Four principal component factor scores were generated and the first factor score was used to define a household wealth index. The total score was categorized in-to low and high scores using the median score as a cutoff point. Favorable conditions categorized in to a higher score of the wealth index.

### Ethical review

The Ethics Review Committee of the Public Health Research and Technology Transfer Support Process at the Regional Health Bureau of southern Ethiopia (Institutional Review Board (IRB)) approved the study. As majority of our study participants were rural dwellers and illiterates, obtaining written consent from them was difficult. Therefore, we obtained an informed verbal consent from all study subjects or their relatives prior to the interview. Enumerators asked the study participants or their relative willingness to provide information. Only when the interviewee responds yes to the question, the interview process continued. The ethics committee (IRB) approved the study including this consent procedure. The principal investigator had access to details of patient records. Enumerators involved in the survey; however, they were not aware of detail information of the study participants from the record review. Personal identifiers of the cases were coded prior to analysis and records (TB registries) were kept in a secure place to maintain the confidentiality of clinical information of cases.

## Results

### Socio-demographic and clinical characteristics

Of 2,730 eligible registered smear-positive TB cases, 2,272 (83.2%) were interviewed, while 458 (16%) were not interviewed due to migration to other areas. There was no baseline difference by age at the time of diagnosis, sex, address and treatment category between the study participants and the loss to follow-up cases. (S1–S2 Tables). We followed the TB patients for 8,780.7 person-years of follow-up. The median (IQR) age of the study participants was 26 (20–38) years. About half (1,125 participants) were male, 1,810 patients (79.7%) were rural dwellers and 742 (32.7%) patients had no formal education. More than 42% of the study participants (965) were farmers, 702 (30.9%) had a family size of at least 6 people, and 702 (30.9%) of the patients had low household wealth (Table 1).

**Table 1:** Baseline and clinical characteristics of TB patients in Dale district, South Ethiopia.

Characteristics		Number	%
Age in years	Median (IQR)	26 (20–38)	
Sex	Male	1,125	49.5
	Female	1,147	50.5
Age group	0–14	161	7.1
	15–34	1,372	60.4
	> 34	718	31.6
	Missing	21	0.9
Address	Rural	1,810	79.7
	Urban	462	20.3
Education	No education	742	32.7
	Formal education	1,425	62.7
	Missing	105	4.6
Family size	1–3 people	733	32.3
	4–5 people	837	36.8
	≥ 6 people	702	30.9
Wealth index	Low score	1,133	49.9
	High score	1,133	49.9
Treatment category	New cases	1,139	50.1
	Re-treatment cases	148	5.9
	Others	14	0.6
	Missing	1	0.0
Treatment outcome	Cured	1,639	72.1
	Treatment completed	250	11.0
	Other#	383	26.6
Treatment times	1 times	1,587	94.0
	2 times	86	5.1
	3–4 times	15	.9
Re-treatment outcome	Cured	53	52.5
	Completed	29	28.7
	Other¤	19	18.8

N.B: IQR = interquartile range;

^#^ Other treatment outcome = persons lost to follow-up TB treatment, died, transferred, treatment failure and unknown;

^¤^ Other re-treatment outcome = died, persons lost to follow-up TB treatment, transferred and unknown

New smear-positive TB cases constituted 2,123 (93.4%) patients. During the initial treatment, 1,889 (83.1%) of the TB patients were successfully treated, while 90 (3.9%) patients died. Of the successfully treated TB cases, 101 (6.0%) patients treated for additional one or more times (Table 1).

### Recurrent TB

Recurrence rate of TB during the study period was 15.2 per 1,000 person-years. The risk of TB recurrence was high for re-treatment TB cases (aHR, 2.7; 95% CI, 1.4–5.3) (Table 2). Nearly three-fourth of TB recurrence occurred during the first 5 years of post-treatment (Table 3).

**Table 2:** Risk factors of TB recurrence among TB patients in Dale district in southern Ethiopia.

Variables	TB recurrence		Person- Years	Death/1,000 P-Y	cHR (95% CI)	aHR (95% CI)
	No	Yes				
All patients	1,688	101	6,645.7	15.2		
Age group**						
0–14 years	129	6	507.9	11.8		
15–34 years	1,005	49	4,395.8	11.1	0.9 (0.4–2.0)	0.9 (0.4–2.2)
> 34 years	446	46	1,703.9	27.0	2.3 (0.9–5.4)	2.1 (0.9–5.1)
**Residence**						
Rural	1,236	83	5,025.7	16.5	1.6 (0.9–2.6)	1.5 (0.9–2.8)
Urban	351	18	1,620.0	11.1		
**Treatment category**						
New	1,488	91	6,314.2	14.4		
Re-treatment	86	10	331.5	30.2	3.1 (1.6–5.9)	2.7 (1.4–5.3)
Other*	13	0	54	-	0.0 (0.0–8.269E+143)	0.0 (0.0–2.248E+170)
Education**						
No education	504	36	2,004.9	18.0	1.4 (0.9–2.1)	1.0 (0.6–1.5)
Formal education	1,011	59	4,359.8	13.5		
**Wealth index**						
Low score	731	54	3,390.7	15.9	1.5 (1.0–2.2)	1.2 (0.8–1.8)
High score	856	47	4,248.9	11.1		

cHR: crude hazard ratio, aHR: adjusted hazard ratio;

**Missing cases: Age = 7 cases, Education = 78 cases;

* Other treatment category = transfer in cases

**Table 3:** TB recurrence and death of TB patients during different periods of follow-up in Dale district, South Ethiopia.

Follow-up time	During treatment	Post-treatment			All
	(Day 1–240)	1^st^ year (Day 241–606)	2^nd^– 5^th^ year (Day 607–2,067)	6^th^– 10^th^ year (Day 2,068–3,893)	Total (Day 1–3,830)
Recurrence, n (%)	-	9 (8.9)	65 (64.4)	27 (26.7)	101 (100)
Death, n (%)	96 (40.3)	48 (20.2)	75 (31.5)	19 (8.4)	238 (100)

### Mortality of TB patients

**Fig 2:**
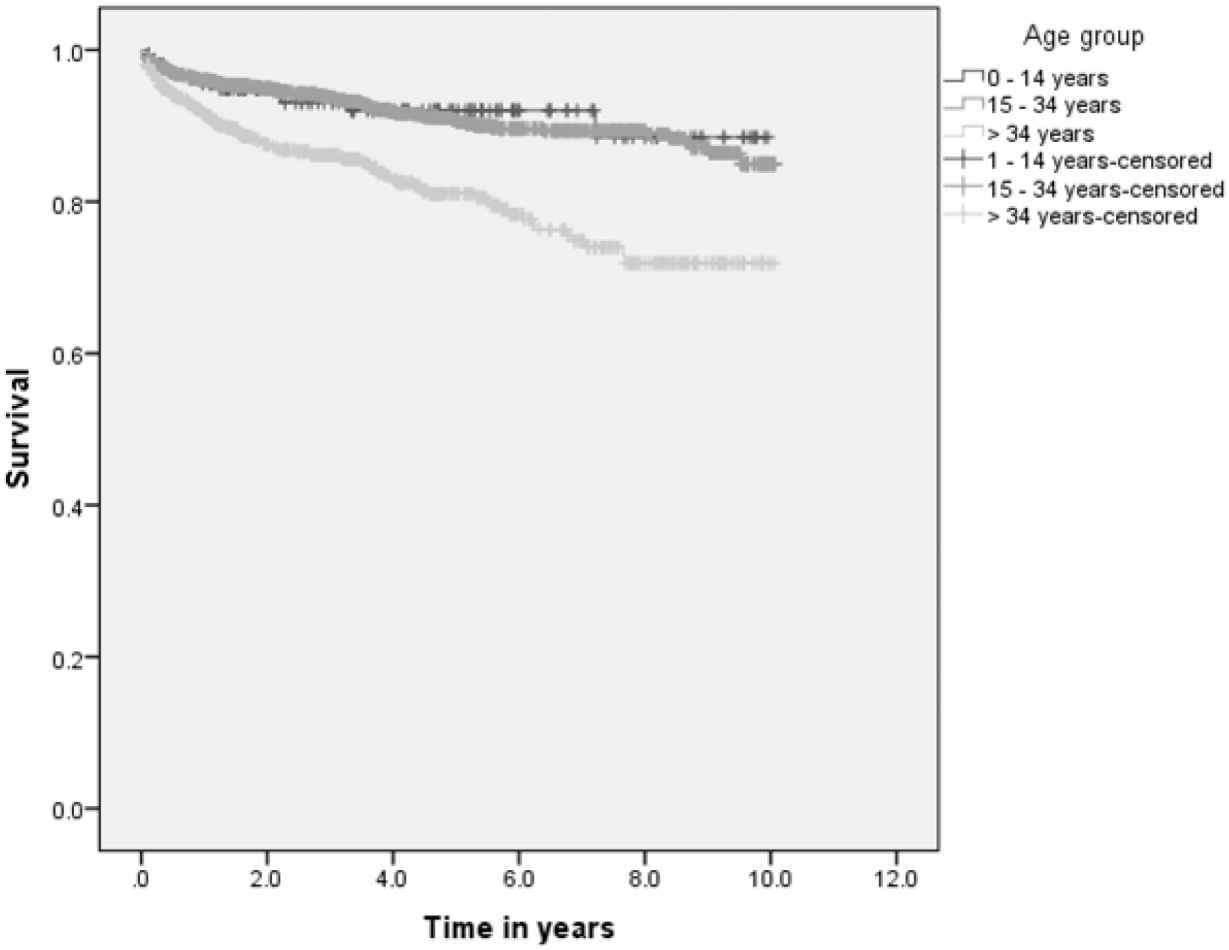
Survival probability of TB patients by age group in Dale district, South Ethiopia.

**Fig 3:**
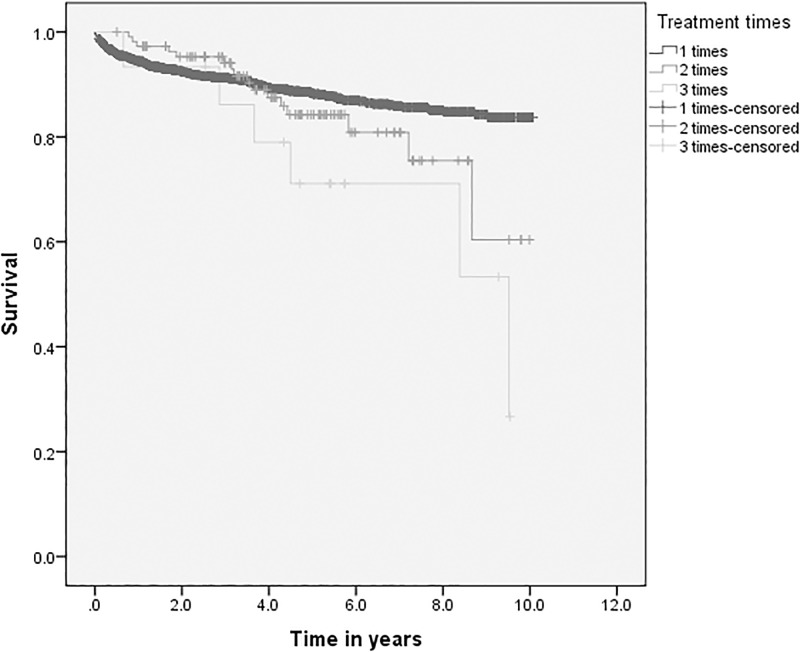
Survival probability of TB patients by treatment times in Dale district, South Ethiopia.

Overall 238 (10.5%) patients died, which gives a mortality rate of 27.1 per 1,000 person-years. About 92% of deaths occurred during treatment and within five years after initiation of treatment (Table 3). The survival probability of TB patients was lowest among patients above 34 years of age, patients with poor treatment outcome and patients treated for three or more times. The survival probability was lower for poor patients (Figs 2 and 3, S1 and S2 Figs).

The excess mortality of TB patients was 4.4/1,000 person-years. Similar measures were 41.2/1,000 person-years for patients aged above 34 years, 20.3/1,000 person-years for male patients and 22.1/1,000 person-years for patients living in urban areas. The overall SMR for TB patients was 1.2 (95% CI; 0.9–1.7). Patients aged below 15 years had the highest SMR 8.0 (95% CI 5.3–11.8). Also male patients 2.8 (95% CI 2.0–3.8) and patients from urban area 6.3 (95%CI 4.4–8.8) had a higher SMR (Table 4).

**Table 4:** Excess mortality among the TB patients in Dale district, South Ethiopia.

Characteristics	Cases	Deaths	Person-years follow-up	Observed deaths (O)	Death rate/year in Sidama zone	Expected deaths (E)	SMR (95%CI)	P-value	Excess mortality/1000 PY
All patients	2,272	238	8,780.7	27.1	0.0100	22.7	1.2 (0.9–1.7)	< 0.001	4.4
Age group*									
0–14 years	161	12	652.8	18.4	0.0139	2.3	8.0 (5.3–11.8)	<0.001	16.1
15–34 years	1372	110	5,683.5	19.4	0.0046	6.3	3.1 (2.1–4.5)	<0.001	13.1
> 34 years	718	113	2346.7	48.2	0.0097	7.0	6.9 (5.3–8.9)	<0.001	41.2
**Sex**									0.0
Male	1,125	137	4,340.8	31.6	0.0100	11.3	2.8 (2.0–3.8)	<0.001	20.3
Female	1,147	101	4,439.9	22.7	0.0120	13.8	1.7 (1.1–2.3)	<0.001	9.0
**Residence**									
Rural	1,810	183	6,688.9	27.4	0.0100	18.1	1.5 (1.1–2.1)	<0.001	9.3
Urban	462	55	2,091.8	26.3	0.0090	4.2	6.3 (4.4–8.8)	< 0.001	22.1

SMR: standardized mortality ratio

CFR was highest during the first five years of post-treatment. Elderly patients and patients with a household size of 1–3 people had the highest CFR. Poor patients and patients with recurrent disease also had a higher CFR (Table 5).

**Table 5:** Case fatality rate of TB patients during different periods of follow-up in Dale district, South Ethiopia.

Characteristics	Population at risk	¤CFR during treatment period		Post-treatment CFR¤		Total
		First 2 months (Day 1–60)	Month 3–8 (Day 61–240)	1^st^– 5^th^ year (Day 241–2,067)	6^th^– 10^th^ year (Day 2,068–3,893)	
		n (%)	n (%)	n (%)	n (%)	n (%)
The cohort	2,272	45 (2.0)	51 (2.2)	123 (5.4)	19 (0.89)	238 (10.5)
Age group*						
0–14 years	161	3 (1.9)	3 (1.9)	5 (3.1)	1 (0.6)	12 (7.5)
15–34 years	1,372	21(1.5)	23 (1.7)	58 (4.2)	8 (0.6)	110 (8.0)
> 34 years	718	21 (2.9)	24 (3.3)	58 (8.1)	10 (1.4)	113 (15.7)
**Sex**						
Male	1,125	20 (1.8)	29 (2.6)	74 (6.6)	14 (1.2)	137 (12.2)
Female	1,147	25 (2.2)	22 (1.9)	49 (4.3)	5 (0.4)	101 (8.8)
**Residence**						
Rural	1,810	38 (2.1)	36 (2.0)	95 (5.2)	14 (0.8)	183 (10.1)
Urban	462	7 (1.5)	15 (3.2)	28 (6.1)	5 (1.1)	55 (11.9)
**Family size**						
1–3 people	733	20 (2.7)	21 (2.9)	49 (6.7)	7 (1.0)	97 (13.2)
4–5 people	837	16 (1.9)	16 (1.9)	41 (4.9)	6 (0.7)	79 (9.4)
≥ 6 people	702	9 (1.3)	14 (2.0)	33 (4.7)	6 (0.9)	62 (8.8)
**Wealth index**						
Low	1,133	28 (2.5)	30 (2.7)	69 (6.1)	8 (0.7)	135 (11.1)
High	1,139	17 (1.5)	21 (1.8)	54 (4.7)	11 (1.0)	103 (9.0)
**Treatment outcome**						
Cure	1,639	3 (0.2)	10 (0.6)	78 (4.8)	15 (0.9)	106 (6.5)
Treatment complete	250	1 (0.4)	1 (0.4)	10 (4.0)	2 (0.8)	14 (5.6)
Other#	383	41 (10.5)	40 (10.4)	35 (9.1)	2 (0.5)	118 (30.8)
**Recurrent TB**						
Yes	101	0 (0.0)	0	15 (14.9)	4 (4.0)	19 (18.8)
No	1,587	0	0	78 (4.9)	12 (0.8)	90 (5.7)

^**¤**^ CFR = Case fatality rate

*Missing cases: Age = 21 cases;

^#^ Other treatment outcome = persons lost to follow-up TB treatment, died, transferred, treatment failure and unknown

Death risk was highest among patients aged above 34 years (aHR, 2.1; 95% CI, 1.2–3.9). The aHR and 95% confidence interval of mortality for patients treated at least 3 times was (4.8; 95% CI, 2.1–11.1) and for patients with poor treatment outcome was (6.7; 95% CI, 5.1–8.9). Patients with low score of household wealth index had a higher risk of death, (aHR, 1.3; 95% CI, 1.0–1.8). Household size showed marginal association with TB death (Table 6).

**Table 6:** Risk factors of death among TB patients in Dale district, South Ethiopia.

Variables	Death		Person-years	Death/1000 PY	cHR (95% CI)	aHR (95% CI)
	No	Yes				
All patients	2,034	238	8,780.7	27.1		
**Sex**						
Male	988	137	4,340.8	31.6		
Female	1,046	101	4,439.9	22.7	0.7 (0.6–0.9)	0.8 (0.6–1.0)
Age group*						
0–14 years	149	12	652.8	18.4		
15–34 years	1,262	110	5,683.5	19.4	1.1 (0.6–1.9)	0.9 (0.5–1.6)
> 34 years	605	113	2346.7	48.2	2.5 (1.4–4.5)	2.1 (1.2–3.9)
**Treatment times**						
1 time	1,931	216	8,211.1	26.3		
2 times	94	16	487.2	32.8	1.3 (0.8–2.1)	1.0 (0.6–1.7)
3–4 times	9	6	82.3	72.9	3.0 (1.4–6.8)	4.8 (2.1–11.1)
**Treatment outcome**						
Cure	1,533	106	6,595.9	16.1		
Treatment complete	236	14	1,128.8	12.4	0.8 (0.5–1.4)	0.8 (0.4–1.4)
Other#	265	118	1,055.9	111.8	6.4 (4.9–8.3)	6.7 (5.1–8.9)
Education*						
No education	659	83	2,700.7	30.7	1.2 (0.9–1.6)	0.9 (0.7–1.2)
Formal education	1,286	139	5,705.0	24.4		
**Family size**						
1–3 people	636	97	2,877.5	33.7		
4–5 people	758	79	3,161.5	25.0	0.7 (0.5–1.0)	0.7 (0.5–1.0)
≥ 6 people	640	62	2,741.6	22.6	0.7 (0.5–0.9)	0.7 (0.5–1.0)
**Wealth index**						
Low score	998	135	3,979.8	33.9	1.5 (1.2–1.9)	1.3 (1.0–1.8)
High score	1,036	103	4,800.8	21.5		

cHR: crud hazard ratio, aHR: adjusted hazard ratio; Missing cases: Age = 21 cases, Education = 105 cases;

^#^ Other treatment outcome = persons lost to follow-up TB treatment, died, transferred, treatment failure and unknown

## Discussion

In this retrospective cohort study, we found high TB recurrence and high mortality among smear-positive TB patients who initiated and completed DOTS. Excess mortality due to TB was observed among male patients, patients from urban areas and patients in the age group of above 34 years. Treatment category predicted TB recurrence, while the risk of death increased among patients above 34 years, patients with poor treatment outcome, patients treated for at least 3 times and among poor patients.

We found lower recurrence rate of TB than the report from Vietnam [9]. However, our finding was higher than the national TB incidence of 177 per 100,000 population in 2016 [1], TB recurrence rate reported in an earlier study [10] and reports from other settings [18, 19]. High TB burden in the study area might have contributed to this finding [10]. In high TB incidence areas, recurrences mostly occur due to re-infection [19, 32]. This is because the risk of re-infection increases with an increase in TB burden. Recurrence after re-infection is a constant risk over time [33]. However, recurrence due to relapse occurs closer to the time of cure [34]. It can be the first year after completion of treatment among our study population, during which 9 TB cases were detected. A study from Barcelona showed that majority of TB recurrence occurred in the first three years after completion of treatment [35]. In our study, about three-fourth of recurrent cases occurred within 5 years after completion of treatment. Therefore, interventions targeted on prevention of transmission and strengthening the TB control program could help in reducing recurrence of TB. More attention should be given during the first five years after treatment completion.

An earlier study from southern Ethiopia reported that, TB recurrence was not associated with age, sex, occupation, marital status and level of education [10]. In the present study, the risk of recurrence among re-treatment TB cases was high and this finding is in agreement with the reports from Spain [19] and elsewhere [35]. So, we suggest follow-up of re-treatment TB cases to identify and treat recurrent TB as early as possible. This may contribute to reducing the TB burden in the study area.

TB death rate in our study was higher than the national death rate, 25 per 100,000 population [1] and the post-treatment death rate reported in South Ethiopia among all forms of TB cases [11]. The proportion of TB cases died during treatment in this study (4.2%) was higher than the report from Addis Ababa and an earlier report in the study area but it was lower than the report from Northwest Ethiopia [2–4]. The reported proportions in all of these studies were for all forms of TB, while we did our study among smear-positive TB cases. In-consistent to the report from other setting [4], among patients who died during treatment, 46.9% died within 2 months after initiation of the treatment. This could be due to an advanced disease caused by delay in diagnosis related to poor access and utilization of health care [36]. Majority of our study participants (79.7%) were rural dwellers and have poor access to TB care since most of the TB diagnosis facilities are located in urban areas. Moreover, rural people have low socioeconomic status and poor knowledge on TB which hinders them from utilizing the service. Therefore, improving access to TB care and improving the socioeconomic status of rural population could reduce the death of TB patients [37].

In our study, highest CFR was observed during the first five years after completion of treatment. This is in agreement with the report from India, where the CFR of TB steadily increased among cured patients from 12 to 48 months [38]. Lung function impairment such as obstructive pulmonary diseases (COPD) might contribute to the death of TB patients during the early years of post-treatment. Studies in various settings have shown that patients treated for TB can develop airflow obstruction. Therefore, assessing the post-treatment lung function of TB patients and delivering appropriate interventions could reduce TB deaths.

In this study, patients with poor treatment outcome had an increased risk of death. In agreement to our finding, some studies reported persons lost to follow-up TB treatment and treatment failure TB cases had the highest risk of death [7, 17]. Patients treated three or more times also had the highest risk of death among our study population. This is in-consistent with the report from other settings [15, 23]. Recurrent TB may increase lung function impairment [5, 6, 39, 40] and this could further increase the risk of death. Therefore, follow-up of re-treatment TB cases and managing complications of TB like COPDs could reduce the risk of death among TB patients.

In this study, we found a higher risk of mortality among the elderly, this may be due to an increased magnitude of comorbidities with age as it was reported in the study by Chou LH et al. [41]. For about 60% of the deaths in our study, the cause of death was other diseases. Besides the DOTS, managing comorbidities among the elderly TB patients could minimize the risk of death.

One of the limitations of our study is unavailability of data by HIV status. So we were not able to estimate the effect of HIV on TB recurrence and death because of the unavailability of HIV status of the patients in unit TB registries. However, the national prevalence of HIV was as low as 2.3% (0.9% in rural areas) [42]. Second, variables like age, education and treatment category had missing values. However, the proportion of missing cases for these variables was very low.

In conclusion, among smear-positive TB patients initiated and completed DOTS, we observed high TB recurrence and death. Compared to the general population, we found higher mortality among the TB patients. Re-treated cases had a higher risk of TB recurrence. Age, treatment outcome, treatment times and wealth index predicted death of TB patients. The excess mortality observed among patients aged above 34 years was high. The high risk of TB recurrence among the re-treated TB cases, the high death rate and the high risk of death among selected groups of TB patients increase the TB burden in the study area. The burden may be even higher having included patients with HIV. Therefore, it is important to give due attention to minimize TB burden in the study area and in other similar settings. Long-term follow-up of TB patients with the risk factors could minimize recurrences through early detection and management. It could also lower the risk of deaths through timely identifying and managing the risk factors. Managing comorbidities and complications of TB could minimize death of TB patients. We recommend further studies to assess the effect of TB on lung function among TB patients in the study area.

## Supporting information

S1 Fig Survival probability of TB patients by wealth index in Dale district, South Ethiopia. (TIFF)

S2 Fig Survival probability of TB patients by treatment outcome in Dale district, South Ethiopia. (TIF)

S1 Table Baseline difference of the study participants and the missing cases for mortality assessment. (DOCX)

S2 Table Baseline difference among the study participants and the missing cases for TB recurrence assessment. (DOCX)
