# Correction: Long-term outcome of smear-positive tuberculosis patients after initiation and completion of treatment: A ten-year retrospective cohort study

**DOI:** 10.1371/journal.pone.0196432

**Published:** 2018-04-19

**Authors:** Mesay Hailu Dangisso, Endrias Markos Woldesemayat, Daniel Gemechu Datiko, Bernt Lindtjørn

The affiliation for the fourth author is incorrect. Bernt Lindtjørn is not affiliated with #1 but with #2 University of Bergen, Faculty of Medicine, Center for International Health, Bergen, Norway.
