# Supplementary figures and images for: Long-term outcome of smear-positive tuberculosis patients after initiation and completion of treatment: A ten-year retrospective cohort study
Source: PLoS One. 2018 Mar 12;13(3):e0193396. doi: 10.1371/journal.pone.0193396 (PMC5846790; doi:10.1371/journal.pone.0193396)

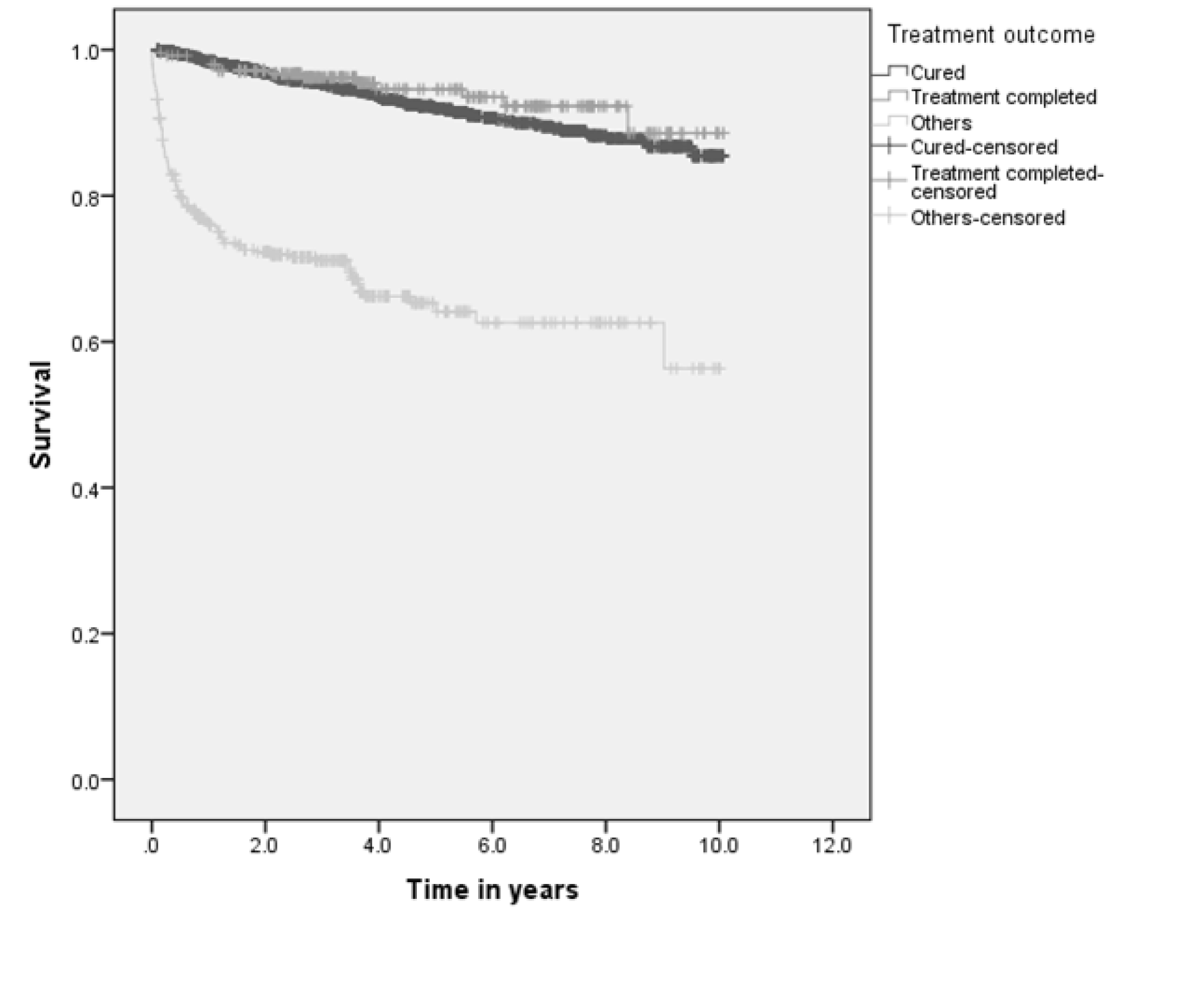

Supplement: S2 Fig — (TIF) [file pone.0193396.s002.tif]
