# Supplementary material for: Long-term outcome of smear-positive tuberculosis patients after initiation and completion of treatment: A ten-year retrospective cohort study
Source: PLoS One. 2018 Mar 12;13(3):e0193396. doi: 10.1371/journal.pone.0193396 (PMC5846790; doi:10.1371/journal.pone.0193396)
Supplement: S1 Table — (DOCX) [file pone.0193396.s003.docx]

**S1 Table. Baseline difference of the study participants and the missing cases for mortality assessment.**

| **Characteristics** | | **Study participants,**  **N^o^ (%)** | **Missing cases, N^o^ (%)** | **P-value** |
| --- | --- | --- | --- | --- |
| Age group in years | 0 – 14 | 161 (7.1) | 32 (7.0) |  |
|  | 15 – 34 | 1,372 (60.4) | 308 (67.2) | 0.6 |
|  | > 34 | 718 (31.6) | 118 (25.8) | 0.2 |
|  | Missing | 21 (0.9) | - | - |
| Sex | Male | 1,125 (49.5) | 216 (52.8) |  |
|  | Female | 1,147 (50.5) | 242 (47.2) | 0.35 |
| Address | Rural | 1,810 (79.7) | 355 (77.5) | 0.3 |
|  | Urban | 462 (20.3) | 103 (22.5) |  |
| Education | No education | 742 (32.7) | - | - |
|  | Formal education | 1,425 (62.7) | - | - |
|  | Missing | 105 (4.6) | - | - |
| Family size | 1 - 3 people | 733 (32.3) | - | - |
|  | 4 - 5 people | 837 (36.8) | - | - |
|  | > 6 people | 702 (30.9) | - | - |
| Wealth index | Low score | 1,155 (50.8) | - | - |
|  | High score | 1,117 (49.2) | - | - |
| Treatment category | New cases | 2,123 (93.4) | 432 (94.3) |  |
|  | Re-treatment cases | 148 (5.9) | 25 (5.5) | 0.4 |
|  | Other** | 14 (0.6) | 1 (0.2) | 0.3 |
|  | Missing | 1 (0.0) |  |  |
| Treatment outcome | Cured | 1,639 (72.1) | 289 (63.1) |  |
|  | Treatment completed | 250 (11.0) | 81 (17.7) | < 0.005 |
|  | Other# | 383 (26.6) | 88 (19.2) | 0.05 |
| Treatment times | 1 times | 1,587 (94.0) | - | - |
|  | 2 times | 86 (5.1) | - | - |
|  | 3 - 4 times | 15 (0.9) | - | - |
| Re-treatment outcome | Completed | 53 (52.5) | - | - |
|  | Cured | 29 (28.7) | - | - |
|  | Others** | 19 (18.8) | - | - |

N.B: ** Other treatment category = transfer in cases; # Other treatment outcome = persons lost to follow-up, died, transferred, treatment failure and unknown
