# Supplementary material for: Long-term outcome of smear-positive tuberculosis patients after initiation and completion of treatment: A ten-year retrospective cohort study
Source: PLoS One. 2018 Mar 12;13(3):e0193396. doi: 10.1371/journal.pone.0193396 (PMC5846790; doi:10.1371/journal.pone.0193396)
Supplement: S2 Table — (DOCX) [file pone.0193396.s004.docx]

**S2 Table. Baseline difference among the study participants and the missing cases for TB recurrence assessment.**

| **Characteristics** | | **Study participants, N^o^ (%)** | **Missing cases, N^o^ (%)** | **P-value** |
| --- | --- | --- | --- | --- |
| Age group in years | 0 – 14 | 135 (8.0) | 26 (7.0) |  |
|  | 15 – 34 | 1054 (62.4) | 248 (67.2) | 0.4 |
|  | > 34 | 492 (29.1) | 95 (25.8) | 0.5 |
|  | Missing | 21 (0.9) | - | - |
| Sex | Male | 834 (49.4) | 194 (52.6) |  |
|  | Female | 854 (50.6) | 175 (47.4) | 0.3 |
| Address | Rural | 1319 (78.1) | 295 (79.9) | 0.5 |
|  | Urban | 369 (21.9) | 74 (20.1) |  |
| Treatment category | New cases | 1579 (93.5) | 350 (94.9) |  |
|  | Re-treatment cases | 96 (5.7) | 18 (4.9) | 0.5 |
|  | Other** | 13 (0.8) | 1 (0.3) | 0.3 |
| Treatment outcome | Cured | 1443 (85.5) | 288 (78.0) |  |
|  | Treatment completed | 245 (14.5) | 81 (22.0) | < 0.005 |

N.B: ** Other treatment category = transfer in cases
